# Healthy Behaviors Associated with Changes in Mental and Physical Strength in Urban African American and White Adults

**DOI:** 10.3390/nu13061824

**Published:** 2021-05-27

**Authors:** Marie Fanelli Kuczmarski, Elizabeth Orsega-Smith, Nicolle A. Mode, Rita Rawal, Michele K. Evans, Alan B. Zonderman

**Affiliations:** 1 Laboratory of Epidemiology and Population Sciences, National Institute on Aging, NIH, 251 Bayview Blvd. Suite 100, Baltimore, MD 21224, USA; nicolle.mode@nih.gov (N.A.M.); evansm@grc.nia.nih.gov (M.K.E.); zondermana@gmail.com (A.B.Z.); 2 Department of Behavioral Health and Nutrition, University of Delaware 26N College Ave, Newark, DE 19716, USA; eosmith@udel.edu (E.O.-S.); rita@udel.edu (R.R.)

**Keywords:** diet, inflammation, handgrip, health behaviors, strength, SF-12

## Abstract

Over time, adherence to healthy behaviors may improve physical and mental strength which is essential for successful aging. A plausible mechanism is the reduction of inflammation. Research on the association of risky health behaviors on change in strength with age is limited. This study examined changes in the inflammatory potential of the diet, smoking, illicit drug use with changes in strength in a racially and socioeconomically diverse adult sample from the Healthy Aging in Neighborhoods of Diversity Across the Life Span study. The dietary inflammatory index (DII) was calculated from 35 food components derived from multiple 24-h dietary recalls. Strength was evaluated by handgrip strength (HGS), SF-12 PCS and SF-12 MCS (physical and mental component scores). Repeated measures analyses were used to examine associations. At baseline, mean age was 48.4 ± 0.25 years, 56% of the sample were women, and 58% African American. Significant 4-way interactions were found between age, race, socioeconomic status, and DII for women, on change in HGS (*p* < 0.05) and in SF-12 PCS (*p* < 0.05) and for men, in change in SF-12 PCS (*p* < 0.05). Improvements in SF-12 MCS were associated with all three health behaviors as main effects. This study provided evidence that changes towards improving healthy behaviors, diet with anti-inflammatory potential, not smoking cigarettes and not using illicit drugs, were associated with improved strength. Health professionals, especially registered dietitians and health coaches, should create lifestyle interventions to reduce inflammation targeting change in more than one risky health behavior.

## 1. Introduction

Strength has been defined as a vital component of healthy aging [1]. It includes two aspects—“body physical”, namely skeletal muscle strength, and mental strength which includes cognitive ability and psychological wellbeing [2]. Establishing and preserving healthy behaviors across the lifespan is essential for mental and physical strength in later years. 

There appears to be cross-communication between physical and mental strength [1]. Contracting skeletal muscle is a source of neurotropic factors which regulate brain synapses [3]; whereas inactivity causes muscle atrophy and increases adiposity [4]. Low muscle strength and mass are associated with brain atrophy and cognitive impairment [3]. Kim and colleagues [5] found that persons 45 to 65 years of age with greater muscle strength had better cognitive function, specifically higher attentional abilities and hippocampus memory performance. In older adults there appears to be a dose-response of direct measures of physical function to physical and mental health [6]. 

It is widely recognized that strength declines with age. Potential mechanisms for this age-related decline in physical and mental strength are low vitamin D levels and inflammaging, a systemic, chronic low-grade inflammation [3,4,7,8]. With age, there is a decreased expression of the intracellular 1,25 di-hydroxyvitamin D receptor on muscle tissue, an increase in intramuscular fat, and reduction of lean skeletal muscle mass. Among women, the loss of estrogen at menopause is associated with increased visceral fat and decreased muscle strength [9]. The increase in body fat with age may result in obesity which is considered an inflammatory state [10]. Inflammaging is characterized by high systemic cytokine and acute phase protein circulation. Vitamin D inhibits inflammation by regulating inflammatory cytokine production and inhibiting the proliferation of proinflammatory cells [11]. Inflammaging has been identified as a common mechanism explaining the complex associations among chronic diseases, mental health disorders, and risky health behaviors [4,12,13]. 

Risky behaviors include but are not limited to consuming a Western dietary pattern, tobacco smoking, sleep disturbance, and physical inactivity [14,15,16,17]. Oftentimes these behaviors cluster or co-occur in population-based studies [18,19] and act synergistically on systematic inflammation [20]. The Western-type dietary pattern can cause metaflammation, a chronic state of metabolic inflammation, resulting in persistent immune system stimulation, gut microbiota dysbiosis, epigenetic modifications, and accumulation of senescent cells [4,21,22,23]. Smoking appears to negatively impact both physical and mental strength [24,25], promoting inflammation via oxidative stress and increased production of inflammatory cytokines [26,27]. The use of illicit drugs such as cocaine can also impact inflammatory cytokines [28,29,30] and may affect strength. One of the most common clusters associated with increased mortality among adults is unhealthful diets and smoking [18].

Even though evidence exists that health behaviors are related in complex ways [20,31], our understanding of how health behaviors relate and collectively predict change in strength over time is limited. Our knowledge of the socio-demographic characteristics that predict risky behavior combinations is also limited [18]. This study will explore changes in physical and mental strength associated with changes in three risky health behaviors using data from the Healthy Aging in Neighborhoods of Diversity across the Life Span Study. The behaviors are the inflammatory potential of the diet consumed, cigarette smoking, and the use of illicit drugs. Our previous research with the HANDLS sample revealed they typically consumed a diet representative of the Western-type pattern. This eating pattern is high in ultraprocessed foods, red meats, refined grains, and simple carbohydrates and low in fruits, vegetables, whole grains, and dietary fiber [32]. We hypothesize that persons of low socioeconomic status with risky behaviors will experience greater decline in strength with age.

## 2. Methods

### 2.1. Healthy Aging in Neighborhoods of Diversity across the Life Span [HANDLS] Study

**Figure 1:**
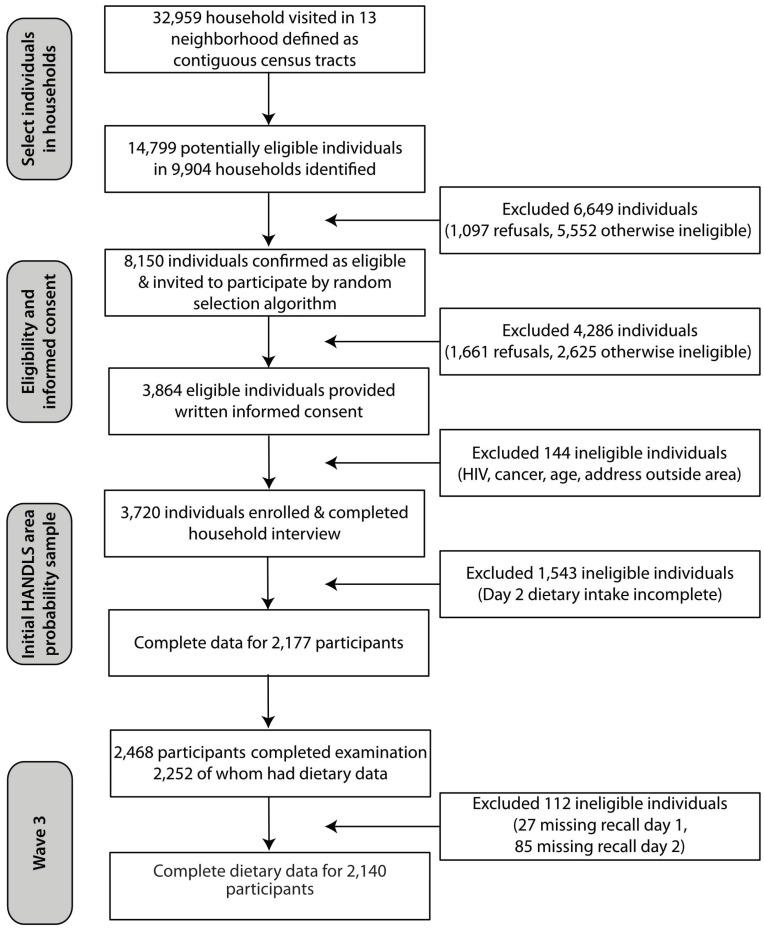
Flow diagram of the Healthy Aging in Neighborhoods of Diversity across the Life Span Study’s household screening, participant eligibility, and response rates.

A detailed description of the HANDLS study, a prospective epidemiological study, is available elsewhere [33,34]. The HANDLS study design was a 4-way factorial cross of age (seven five-year age bands from 30 through 64 years), sex (men and women), race (self-reported; non-Hispanic African American and non-Hispanic White), and poverty status (self-reported household income < 125% or >125% of the 2004 Health and Human Services poverty guidelines [35]). Figure 1 provides a flow diagram of the study’s household screening, participant eligibility, and response rates. Participants were drawn from 9904 households in 13 Baltimore City pre-determined neighborhoods that were selected specifically to enable recruitment of men and women in the target racial, poverty status, and age groups. There were 8150 individuals confirmed as eligible and invited to participate in the HANDLS study by random selection algorithm [36]. Exclusion criteria were pregnancy and active cancer treatment within the last 6 months [34]. 

The baseline study initiated in 2004 and completed in 2009, is referred to visit (v) 1 in this article. The first follow-up wave, v 2, was conducted during 2009–2013. For each visit, data collection was conducted in two phases. For v 1, Phase 1 took place in the home and included household screening, recruitment, and completion of questionnaires with each participant (maximum 2 per household). Phase 2 took place in a Mobile Research Vehicle (MRV) stationed in the neighborhood, where participants underwent comprehensive health examinations and completed computer-assisted questionnaires [34]. For v 2, Phase 1 was completed in MRV and Phase 2 by telephone interviews, or both phases were completed at home for those who had difficulty ambulating independently [37].

### 2.2. Sample

Participants were from the baseline and first follow-up visits of the HANDLS study (*n* = 3720). Written consent was obtained from all participants at each wave after they reviewed the laymen’s protocol booklet and watched a video describing all procedures. All participants were compensated with money. The Human Institutional Review Boards at MedStar Health Research Institute, the National Institute of Environmental Health Sciences, National Institutes of Health, and the University of Delaware approved the HANDLS study protocol.

### 2.3. Dietary Methods and Dietary Inflammatory Index [DII]

For all visits, trained interviewers, using the US Department of Agriculture (USDA) Automated Multiple Pass Method (AMPM) [38,39], collected food intake on 2 days. This computer-assisted method and portion estimation visual aids are described elsewhere [40]. A 3-day training and periodic refresher trainings were completed by all interviewers. For v 1, both interviews were done in-person; at v 2, the first recall was done in-person and the second recall was done by telephone. Of the 3720 baseline participants, 2177 adults completed both 24-h recalls at v 1 and 2140 persons completed both recalls at v 2. Quality control evaluation was performed on all recalls and those who reported fasting or consuming less than 500 kcal were considered unreliable and deleted. Dietary recall data were coded with unique food codes using the USDA Food and Nutrient Database for Dietary Studies (FNDDS), 3.0 (2005–2006) for v 1, and FNDDS, 5.0 (2009–2010) for v 2 and Survey Net Coding software [41]. Flavonoid intakes were generated using the 2007–2010 USDA database of flavonoid values [42].

The DII quantifies the overall effect of diet on inflammatory potential and was calculated using 35 of the 45 possible parameters identified by Shivappa and colleagues [43]. They reported that dropping from the maximum 45 to 28 parameters does not decrease in the predictive capability of the DII [43]. This indicates that missing food parameters in the present study would not have a major impact on the scoring. The parameters included energy, alcohol, protein, carbohydrate, dietary fiber, total fat, saturated fat, monounsaturated fat, polyunsaturated fat, omega 3 fatty acids, omega 6 fatty acids, cholesterol, 11 vitamins, 4 minerals, 6 flavonoid classes, caffeine, and tea. The 10 parameters excluded because they were not included in the USDA databases were trans fatty acids, garlic, ginger, onion, pepper, rosemary, saffron, thyme/oregano, turmeric, and eugenol. The greater the DII score the more pro-inflammatory the dietary pattern.

The DII was validated with inflammatory biomarkers [15,44,45,46] and has been shown to be associated with handgrip strength, mental health disorders, and function, namely sarcopenia and frailty [12,47,48,49,50,51]. Using participants in the Melbourne Collaborative Cohort Study, Hodge and colleagues [52] reported that the DII provided similar associations with cardiovascular disease mortality as the Mediterranean Diet Score.

Applying the global composite database to data from the HANDLS study at v 1 and v 2, the possible maximal pro-inflammatory DII score was +10.44 and the maximal anti-inflammatory DII score was −10.44. For the analytical sample, the median was 3.39, the maximal pro-inflammatory score was 7.67, and the maximal anti-inflammatory score was −5.798. The median DII for the men and women in our sample was 2.94 and 3.61, respectively.

### 2.4. Handgrip Strength

Handgrip strength [HGS] is a common biomarker for age-related changes in physical strength [53,54,55,56,57,58]. It is useful as a diagnostic tool for identifying the risk for poor health in older adults because it is reliable, easily applicable, and cost-effective [56].

Trained technicians using the Jamar Hydraulic Hand Dynamometer (Patterson Medical Holdings Inc., Bolingbrook, IL, USA) assessed handgrip strength [59]. This dynamometer registers the maximum kilograms of force per trial. The participants were seated with the elbow of the tested side resting on a table at approximately 160°. Two trials were performed for both the right and left hands with a 15–20 s rest between trials. The test was not performed if the participant reported surgery within the past three months or if they had pain and/or arthritis that would impede their ability to successfully complete the handgrip test. This study used the average force of the two trials for the dominant hand. The right-hand measure was used for those who reported that they were ambidextrous.

### 2.5. Medical Outcomes Study Short Form (SF)-12 Physical and Mental Component Scores [SF-12 PCS and SF-12 MCS, respectively]

The SF-12 is a measure of health adopted from the SF-36 that assesses 8 different domains. SF-12 PCS includes physical functioning, role-physical, bodily pain, and general health while the SF-12 MCS includes scales for vitality, social functioning, role-emotional, and mental health. The individual SF-12 questions are on a five-point Likert scale (e.g., all of the time, most of the time, some of the time, a little of the time, and none of the time) that assess mental health, vitality, and social functioning. The SF-12 questions about pain were on a similar five-point Likert scale (e.g., extremely, quite a bit, moderately, a little bit, not at all). Physical and mental health component *t* scores are linearly transformed scores to have a mean of 50 and a standard deviation of 10 in the general US population [60]. Higher scores indicate better health in addition to less dysfunction and impairment.

### 2.6. Statistical Analyses

Data were distributed continuously and were not significantly skewed. Sex differences in sample descriptive statistics were compared by *t*-tests for means and chi-square tests for categorical measures. Separate repeated measures analyses on changes in HGS, SF-12 PCS, and SF-12 MCS were performed by mixed-effects regression [61] stratified by sex. Mixed-effects regression analyses are preferred for examining data with different numbers of repeated outcome measurements obtained at nonuniform intervals. With time indexed as age, the model computes rate of change for the entire group and then examines individuals’ deviations from the group rate. Mixed-effects regression evaluates the unique effects of individual predictors adjusted for all other predictors in the model, includes both fixed and random effects, accounts for the correlation among repeated measurements on the same participant, and is unaffected by randomly missing data. Mixed-effects regression results are interpreted as the rate of change in the outcome measures as a function of one unit change in the dietary inflammatory index and all relevant covariates. In this study, the analyses examined the influences of changes in DII, current illicit drug use, current cigarette use, and age as the time-varying measures centered at 50 years (*Age_50_*) in decade units, and race and SES as time-invariant measures. Age was centered at 50 years in decade units to facilitate its interpretation relative to the approximate mean age of the sample. Illicit drugs included marijuana, cocaine, and heroin. SES was based on the level of education attained or income, with high SES defined as household income >125% poverty or ≥12 years of education at enrollment. Curvilinear change in outcomes was examined by linear and quadratic age centered at 50 in decade units. Interactions of interest were 3-way and 4-way combinations of linear and quadratic age by SES, race, and DII including 2-way interactions. Mixed-effects regression interactions are interpreted in similar fashion to ordinary regression such that the outcome is a function of the combination of variables included in the interaction term. *p* < 0.05 were considered statistically significant. All analyses were performed using R 4.0.3 [62].

## 3. Results

### 3.1. Sample Characteristics

The mean (± SE) age of the sample at v 1 was 48.4 ± 0.25 years. At v 2, the mean (±SE) increase was 4.58 ± 0.03 years. As shown in Table 1, approximately 56% of the sample were women and 58% of both sexes were African American. There were no differences in the portion of African Americans or socioeconomic status within the sexes. Women were more likely to not smoke or use illicit drugs compared to men. Women reported a diet with significantly higher pro-inflammatory potential than men in this study. Women also had significantly lower HGS and scores for both physical and mental health.

### 3.2. Association of Health Behaviors with HGS

**Figure 2:**
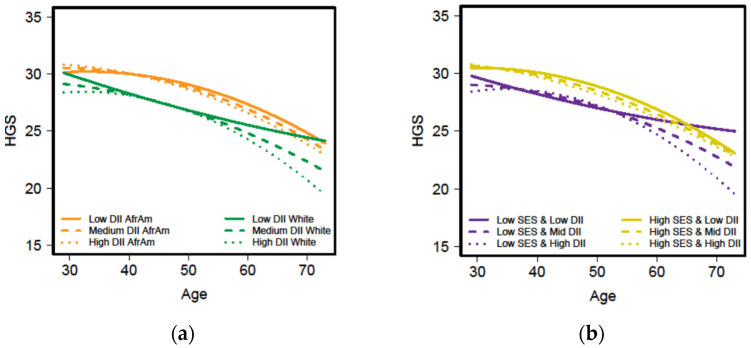
Handgrip strength (HGS) by age for HANDLS women across three levels of the dietary inflammatory index (DII) and either race ((**a**) African American (AfrAM) and White) or socioeconomic status ((**b**) SES). HANDLS-Healthy Aging in Neighborhoods of Diversity across the Life Span.

The regression analyses for women revealed one 4-way and one 3-way significant interactions involving the inflammatory potential of the diet with the change in HGS with age as the outcome (Table S1). The significant 4-way interaction was between *Age_50_ ^2^ x* race (African American vs. White) *x* SES (high vs. low) *x* DII_average_ (β = −1.12, *p* < 0.05). Figure 2a shows the change in handgrip with age by race and the inflammatory potential of the diet while Figure 2b, depicts the relationship by SES and the inflammatory potential of the diet. The DII is illustrated at three levels based on representative values of the DII distribution for this sample. In the figures, low DII represents the lowest quartile values (DII ≤ 1.731) and high DII by the highest quartile values (DII ≥ 4.655). Medium DII represents the values between low and high where median = 3.389. The 4-way interaction suggests a greater decline in HGS over time for African American women, women consuming a diet of high inflammatory potential and of low SES compared to White women, women whose diet had less inflammatory potential and were of high SES, respectively. The decline in HGS appears more accelerated after age 60 (Figure 2a,b). Among men, we found a significant association for change in HGS over time in only one behavior—not smoking cigarettes. In both men and women, not smoking cigarettes was directly associated with the change in HGS with increasing age (β = 1.62, *p* < 0.01 and β = 0.82, *p* < 0.05, respectively) (Table S2).

### 3.3. Association of Health Behaviors with SF-12 PCS

**Figure 3:**
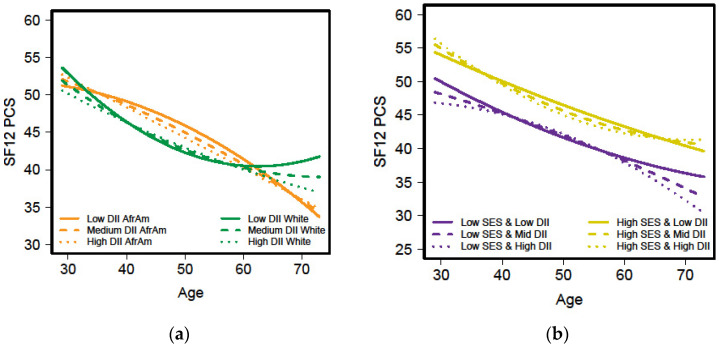
Short Form-12 Physical Component Scores (SF-12 PCS) by age for HANDLS.women across three levels of the dietary inflammatory index (DII) and either race ((**a**) African American (AfrAm) and White) or socioeconomic status ((**b**) SES). HANDLS-Healthy Aging in Neighborhoods of Diversity across the Life Span.

Similar to HGS, the regression analyses for women revealed a significant 4-way interaction between *Age_50_ ^2^ x* race (African American vs. White) *x* SES (high vs. low) *x* DII_average_ with the change in SF-12 PCS with age as the outcome (Table S1; β = −1.56, *p* < 0.05). Figure 3a shows the change in SF-12 PCS with age by race and the inflammatory potential of the diet while Figure 3b, depicts the relationship by SES and the inflammatory potential of the diet. This interaction suggests a greater decline in SF-12 PCS over time for White women compared to African American women (Figure 3a). The change in SF-12 PCS over time across DII groups appears to be similar (Figure 3a,b). Over time, the SF-12 PCS scores were greater for the high SES women compared to the low SES group for all DII groups (Figure 3b).

**Figure 4:**
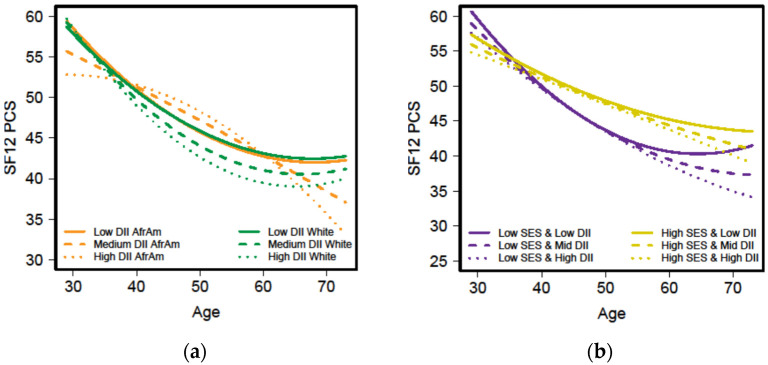
Short Form-12 Physical Component Scores ((SF-12 PCS) by age for HANDLS men across three levels of the dietary inflammatory index (DII) and either race ((**a**) African American (AfrAm) and White) or socioeconomic status ((**b**) SES). HANDLS-Healthy Aging in Neighborhoods of Diversity across the Life Span.

For men, the regression analyses revealed a 4-way, as well as 3-way, and 2-way interactions which included DII, and two main effects [DII and smoking] (Table S2). The significant 4-way interaction for men was between *Age_50_ ^2^ x* race (African American vs. White) *x* SES (high vs. low) and DII_average_ with the change in SF-12 PCS with age as the outcome (Table S2, β = 1.50, *p* < 0.05). Figure 4a shows the change in SF–12 PCS with age by race and the inflammatory potential of the diet while Figure 4b, depicts the relationship by SES and the inflammatory potential of the diet. The decline in SF–12 PCS over time among men appears to be greater for African Americans, those with low SES and men consuming a diet with high inflammatory potential compared to White men, those of high SES, and those who consume a diet with less inflammatory potential (Figure 4a,b). Similar to HGS, not smoking cigarettes was directly associated with increases in SF-12 PCS with increasing age in both sexes (men: β = 2.84, *p* < 0.001 and women: β = 1.39, *p* < 0.05) (Table S2).

### 3.4. Association of Health Behaviors with SF-12 MCS

The regression model for women revealed changes in two health behaviors, not smoking cigarettes (β = 1.48, *p* < 0.05) and not using illicit drugs (β = 1.72, *p* < 0.05), were significantly associated with better mental health over time (Table S1). Among men, changes in food intake to a diet with more anti-inflammatory potential (β = −1.49, *p* < 0.05) and not using illicit drugs (β = 1.41, *p* < 0.05) were significantly associated with enhanced mental health with increasing age (Table S2).

## 4. Discussion

The study findings reflect the multi-dimensionally of strength. The relationships between SES and change in lifestyle behaviors with the change in strength over time were complex and sometimes varied by sex. SES interacted with diet-related behavior to predict physical strength change over time. To our knowledge, this is the first study to report the inflammatory potential of the diet consumed by the HANDLS sample and how it impacted their physical and mental strength. The association of diet with changes in physical strength was not in the same direction for men and women. Improvement in mental strength over time was significantly associated with diet change towards one of more anti-inflammatory potential but only in men. Among both sexes, not smoking cigarettes had a positive direct effect on HANDLS study participants’ physical strength measured by handgrip, while not using illicit drugs was associated with improvements in mental health.

Evidence for the clustering of health behaviors and their association with SES was provided in this study. The greatest decline in physical strength among the women in the HANDLS study sample was the combination of low SES with the consumption of a diet with greater pro-inflammatory potential. While diet did not appear to predict change in the mental health of the women, smoking was associated with greater decline in both mental and physical strength of women. Among men, greater decline in mental health was associated with a diet with higher DII scores (more pro-inflammatory) and use of illicit drugs. The clustering of health behaviors and the association of SES with engagement in multiple behaviors is consistent with the findings in the review by Meader and colleagues [18]

### 4.1. Physical Strength

Physical strength, measured by HGS, was significantly greater in men than in women. Even though the absolute HGS is greater in men compared to women, Perna and colleagues [63] found that the relative decrease from peak physical strength was less in women compared to men when analyzing cross-sectional data from the National Health and Nutrition Examination Survey 2011-12. Our findings revealed that HGS of women was highest at younger ages which correspond to the reported peaks between 30 to 39 years [63]. Then there was a gradual decline in HGS until around age 60 years when the slope became steeper (Figure 2). Using longitudinal SF-12 PCS scores, we found the decline in physical strength for women was less than that of men. Among women, those who were White and categorized as high SES had greater HGS than those who were African American and categorized as low SES.

Additionally, the mean SF-12 PCS scores for men were significantly higher than for women. Assari and colleagues [64] who studied only African American adults, also found men had higher scores than women. They reported that in their economically disadvantaged sample, older age was associated with better SF-12 PCS scores for only African American men. Our study found that among African American men who consumed a diet with less inflammatory potential (i.e., a healthier diet), the change in SF-12 PCS improved with increasing age. In contrast, the inflammatory potential of the diet was only associated with the change in HGS for women.

Across the life span, adherence to eating plans of high diet quality, such as the Mediterranean and DASH (Dietary Approaches to Stop Hypertension) diets, is associated with strength [65,66,67,68] and decreased risk of all-cause mortality [69]. These diets are rich in fruits, vegetables, whole grains, nuts, and protein sources emphasizing seafood, low-fat dairy foods, and lean meats and are considered anti-inflammatory [45,70,71]. With age, in addition to the consumption of high-quality protein, physical activity is an anabolic stimulus for physical strength [72] and have implications for the prevention and treatment of sarcopenia [68,73].

The observed positive direct effect of not smoking cigarettes on HANDLS study participants’ HGS over time is supported by findings of other researchers [24,25]. Compared to nonsmokers, Petersen and colleagues [74] found that among smokers the rate of muscle protein synthesis was lower and the expression of myostatin, a muscle growth inhibitor, was increased. Smoking appears to increase the risk of sarcopenia with age [75,76] and its effect on lowering the strength of muscle has been linked to increased mortality [77]. Free radicals, which cause lipid peroxidation and protein oxidation, are generated by tobacco smoke, and probably cause these effects [78].

### 4.2. Mental Strength

The improvement in mental strength, assessed by the SF-12 MCS, over time was significantly associated with diet in only men. A diet with more anti-inflammatory potential was beneficial. Unlike physical strength, there were no interactions with DII and race or SES. Improvements in mental strength was also associated with not smoking cigarettes among women and not using illicit drugs among both sexes. This finding is supported by research that documents the positive association between nicotine and drug dependence with mental health disorders [79,80].

### 4.3. Potential Mechanisms to Explain Decline in Strength

A diet with greater pro-inflammatory potential and smoking can cause chronic inflammation which could explain the decline in strength of HANDLS study sample with age. The use of illicit drugs is also associated with inflammation suggesting a possible cause for the decline in mental strength observed.

The median DII for the men and women in our sample was indicative of a pro-inflammatory eating pattern. Diet plays a critical role in the regulation of inflammation even with the control of potential confounders [71,81,82]. Zhang and colleagues [83] found a dose-related positive association between energy from ultraprocessed foods and inadequate cardiovascular health metrics among US adults. Previous research with the HANDLS study sample revealed that eating patterns that are more consistent with the Healthy Eating Index and DASH diet quality indices, even though they were suboptimal quality, were associated with lower 10-year cardiovascular risk [59]. Similarly, the results of this study indicate that even small improvements in diet towards anti-inflammatory potential may improve physical and mental strength.

### 4.4. Implications

A decrease in strength over the life span has also been documented through HGS and self-reported data. The SF-12 PCS and SF-12 MCS have been shown to decrease significantly with higher age [84]. These scores are not only linked to strength but also to predictions of morbidity and mortality in older adults. The United States (US) Longitudinal Study of Aging also found that self-assessments of physical, mental, and general health predicted functional decline and mortality [85]. In another US study, lower scores on the SF-12 predicted higher rates of hospitalizations and mortality in community-dwelling older adults [86]. It seems that preventing the decline in strength may improve quality of life in later years.

With aging, strength is essential to health, so minimizing chronic low-grade inflammation seems like a reasonable goal for the general population. Food is considered a fundamental prerequisite for longevity and wellbeing contributing over 26,000 biochemicals [87]. The use of the DII by nutritionists and dietitians may be beneficial when developing dietary interventions for clients. Clients with scores indicative of pro-inflammatory diets could be directed to more plant-based dietary patterns such as the Mediterranean or DASH eating patterns. Since risky behaviors like smoking and alcohol misuse cluster with unhealthful diets, diets low in fruit and vegetable intake [18], health professionals should assess these behaviors when evaluating dietary intake.

The HANDLS study sample is a vulnerable group experiencing low SES and heath inequities. Approximately 49% of the HANDLS study sample smokes cigarettes compared to 21.80% of the US population and 24.02% in other high-income countries across the world [88]. The use of illicit drugs is prevalent in the US [89] with roughly 16% of the study sample reporting to be a current user. Comorbidity patterns exist between mental health disorders and the use of tobacco, illicit drugs, and alcohol in the general population of high-income countries worldwide [79,80]. The dependence on these substances is associated with mental health disorders including depression and anxiety [79]. Smoking cessation along with change in other behaviors that promote positive mental health can improve quality of life [90,91] The development and implementation of targeted lifestyle behavior intervention plans by health professionals could lead to improved overall health and quality of life.

### 4.5. Strengths and Weaknesses of Study

One positive aspect of this study was the use of longitudinal data with a relatively large sample to explore associations. Additionally, food intake was collected using a 24-h recall on two separate days at v 1 and at v 2. The sample represents a vulnerable group that is underrepresented in the nutrition literature and offers new insights into their health behaviors. The mean HGS, SF-12 PCS and MCS values for the HANDLS study sample were slightly lower than estimates reported for the US nationally representative sample [56,63,92] (Table S3). Lastly, strength was addressed by a variety of validated measures, with handgrip recognized as both a biomarker for aging as well as muscle strength.

There are also limitations. Statistical analyses were limited by the relatively short intervals between repeated measures with too few repeats to assess whether there were higher order nonlinear associations between independent and dependent variables. The collection of data on physical activity and sleep began in v 2 and continued into the next visit (v 3), limiting our ability to include these variables in the regression models. In the future when the DII for v 3 becomes available, other analyses can be performed, expanding the time period from the baseline visit to approximately 10–12 years. Perhaps stress, another health-related behavior, was a possible confounder but was not explored in this study. Another limitation is that self-reported dietary intakes are subject to measurement errors. These errors include underreporting of food and beverage intake [93,94] and social desirability bias [95]. While the mental and physical component scores from the SF-12 are considered meaningful measures, they are also self-reported and thus subject to bias. The findings reflect the diverse urban sample studied and are not representative of the entire US population.

## 5. Conclusions

Strength improvements with increasing age are important for overall quality of life and health. The findings of this study illustrate the complexities of the interrelationships among race, SES, and health behaviors with the change in strength with increasing age. All three investigated behaviors, namely diet, smoking cigarettes, and use of illicit drugs, showed significant effects on changes in strength with increasing age. Additionally, sex differences exist in both objective and subjective measures of strength. Consistent with the reports of others, women in the HANDLS sample generally had lower strength than the men [63,64,92,96]. This study suggests changes in personal lifestyle behaviors that promote health may improve strength. The plausible mechanism is the reduction of pro-inflammatory biomarkers, which requires further exploration.

## Figures and Tables

**Table 1:** Characteristics of HANDLS study sample at Visit 1, 2004–2009.

Predictors and Outcome Measures	Overall	Sex		
	*N* = 1907	Men *N* = 839	Women *N* = 1068	*p*
Age, years, X ± SE	48.38 ± 0.21	48.31 ± 0.32	48.43 ± 0.29	0.789
Race, African American, %	58.0	58.5	57.6	0.715
Socioeconomic status, High ^a^, %	81.8	81.9	81.7	0.984
Dietary Inflammatory Index ^b^, X ± SE	3.35 ± 0.05	3.05 ± 0.08	3.58 ± 0.06	<0.001
Cigarette smoker, Not current, %	51.2	45.2	55.9	<0.001
Illicit drug use, Not current, %	82.0	76.4	86.3	<0.001
Handgrip strength, X ± SE	34.45 ± 0.25	42.24 ± 0.35	28.03 ± 0.21	<0.001
SF-12 MCS ^c^, X ± SE	49.56 ± 0.26	50.38 ± 0.36	48.90 ± 0.36	0.004
SF-12 PCS ^c^, X ± SE	47.39 ± 0.26	48.13 ± 0.37	46.80 ± 0.35	0.010

Abbreviations: HANDLS—Healthy Aging in Neighborhoods of Diversity across the Life Span, SF-12 MCS—Short Form-12 Mental Component Summary score, SF–12 PCS–Short; Form–12 Physical Component Summary score. ^a^ High socioeconomic status meant either the household income was above 125% 2004 HHS, poverty guidelines [35] or person had at least a high school education. ^b^ Dietary Inflammatory Index was calculated based on Shivappa et al. method [43] ^c^ Component Scores are represented as t-scores with a mean of 50 and a standard deviation of 10.

## Data Availability

Data are available upon request to researchers with valid proposals who agree to the confidentiality agreement as required by our Institutional Review Board. We publicize our policies on our website https://handls.nih.gov. Requests for data access may be sent to Alan Zonderman (co-author) or the study manager, Jennifer Norbeck, at norbeckje@mail.nih.gov.

## Supplementary Materials

The following are available online at https://www.mdpi.com/article/10.3390/nu13061824/s1, Table S1: Estimated coefficients of change in health behaviors with strength measures in HANDLS women, Table S2: Estimated coefficients of change in health behaviors with strength measures in HANDLS men, Table S3: Comparison of Mean Strength Measures of HANDLS study sample with US nationally representative sample stratified by age and sex [56,92].
