# Supplementary material for: Healthy Behaviors Associated with Changes in Mental and Physical Strength in Urban African American and White Adults
Source: Nutrients. 2021 May 27;13(6):1824. doi: 10.3390/nu13061824 (PMC8226642; doi:10.3390/nu13061824)
Supplement: Supplementary file 1 [file nutrients-13-01824-s001.zip › nutrients-1161991-supplementary.pdf]

Supplementary Tables

Table S1. Estimated coefficients of change in health behaviors with strength measures in HANDLS women

Variables	HGS	SF-12 PCS	SF-12MCS
Intercept	24.04***	35.23***	39.50***
Age_{50}	-0.24	-1.08	1.06
Age_{50}^2	1.79	3.62	0.31
Race, AA	4.64	10.74**	5.56
SES, High	4.61*	11.81***	6.20
$DII_{average}$	0.41	1.26	0.28
Cigarette smoker, Not current	0.82*	1.39*	1.48*
Illicit Drug Use, Not current user	0.19	0.09	1.72*
Age_{50} x SES, high	-1.61	-2.02	-0.08
Age_{50} x Race, AA	-0.81	-2.97	-0.48
Age_{50} X $DII_{average}$	-0.29	-0.07	-0.02
Race, AA x SES, high	-4.35	-11.56**	-3.09
SES, high X $DII_{average}$	-0.87	-2.07**	-0.70
Race, AA X $DII_{average}$	-0.67	-2.16*	-0.70
Age_{50}^2 x SES, high	-2.64	-3.77	0.20
Age_{50}^2 x Race, AA	-2.51	-5.27	1.95
Age_{50}^2 X $DII_{average}$	-0.65	-1.01*	0.74
Age_{50} x SES, high X Race, AA	1.53	4.09*	-0.11
Age_{50} x SES, high x $DII_{average}$	0.21	-0.00	-0.05
Age_{50} X Race, AA x $DII_{average}$	0.05	-0.00	0.10
Race, AA x SES, high x $DII_{average}$	1.11	2.76**	1.09
Age_{50}^2 x Race, AA x SES, high	3.23	5.09	-1.88
Age_{50}^2 x SES, high x $DII_{average}$	0.86*	1.37*	-1.00
Age_{50}^2 x Race, AA x $DII_{average}$	0.80	1.31	-0.96
Age_{50}^2 x Race, AA x SES, high x $DII_{average}$	-1.12*	-1.56*	1.01

*p<0.05, **p<0.01, ***p<0.001

Abbreviations: AA- African American, Age_{50} – age centered at 50 years in decade units, DII – Dietary Inflammatory Index, HANDLS- Healthy Aging in Neighborhoods of Diversity across the Life Span, SES, high- Socio-Economic Status, high defined as either the household income was above 125% 2004 HHS poverty guidelines [35] or education ≥ 12 years

Table S2. Estimated coefficients of change in health behaviors with strength measures in HANDLS men

Variables	HGS	SF-12PCS	SF-12 MCS
Intercept	47.20***	45.66***	50.72***
Age_{50}	-2.14	-3.67	-2.22
Age_{50}^2	-2.53	0.91	-1.22
Race, AA	1.13	-5.67	-3.96
SES, High	-2.33	.2.27	-2.47
$DII_{average}$	-1.39	-1.75**	-1.49*
Cigarette smoker, Not current	1.62**	2.84***	0.25
Illicit Drug Use, Not current user	0.01	-0.89	1.41*
Age_{50} x SES, high	-0.63	0.80	2.97

Age_{50} x Race, AA	0.56	-1.10	2.91
Age_{50} X $DII_{average}$	-0.36	-0.04	0.15
Race, AA x SES, high	0.78	4.27	5.68
SES, high X $DII_{average}$	0.62	1.30	1.18
Race, AA X $DII_{average}$	0.18	3.38***	1.63
Age_{50}^2 x SES, high	1.45	-0.27	1.08
Age_{50}^2 x Race, AA	-0.49	2.62	2.71
Age_{50}^2 X $DII_{average}$	0.29	0.48	-0.01
Age_{50} x SES, high X Race, AA	0.16	0.70	-2.63
Age_{50} x SES, high x $DII_{average}$	0.38	0.15	-0.13
Age_{50} X Race, AA x $DII_{average}$	-0.03	0.27	-0.37
Race, AA x SES, high x $DII_{average}$	-0.11	-2.83**	-1.39
Age_{50}^2 x Race, AA x SES, high	0.87	-1.99	-2.55
Age_{50}^2 x SES, high x $DII_{average}$	-0.14	-0.61	0.16
Age_{50}^2 x Race, AA x $DII_{average}$	-0.22	-1.66**	-0.14
Age_{50}^2 x Race, AA x SES, high x $DII_{average}$	-0.04	1.50*	0.06

*p<0.05, **p<0.01, ***p<0.001

Abbreviations: AA- African American, Age_{50} – age centered at 50 years in decade units, DII – Dietary Inflammatory Index, HANDLS- Healthy Aging in Neighborhoods of Diversity across the Life Span, SES, high- Socio-Economic Status, high defined as either the household income was above 125% 2004 HHS poverty guidelines [35] or education ≥ 12 years

Table S3. Comparison of Mean Strength Measures of HANDLS study sample with US nationally representative sample stratified by age and sex [56,63].

	**HGS**		**SF-12PCS**		**SF-12 MCS**	
	**HANDLS**	**US**	**HANDLS**	**US**	**HANDLS**	**US**
Men						
30-39 years	48.3	47.2	52.4	53.5	50.4	52.1
40-49 years	43.1	45.5	46.4	51.7	48.0	51.8
50-59 years	38.2	42.1	42.8	49.4	48.5	51.6
60-69 years	36.0	38.9	43.0	47.3	47.7	53.2
Women						
30-39 years	30.5	30.0	47.7	52.4	46.4	49.2
40-49 years	26.0	29.4	45.6	50.6	45.5	48.9
50-59 years	24.0	27.5	41.1	48.2	47.0	49.8
60-69 years	22.4	25.3	41.7	45.7	50.4	51.5

Abbreviations: HGS- handgrip strength, SF-12 PCS- Short Form-12 Physical Component Scores, SF-12 MCS- Short Form-12 Mental Component Scores, HANDLS- Healthy Aging in Neighborhoods of Diversity across the Life Span, US- United States
